# Diffusion tensor imaging of the roots of the brachial plexus: a systematic review and meta-analysis of normative values

**DOI:** 10.1007/s40336-020-00393-x

**Published:** 2020-10-09

**Authors:** Ryckie G. Wade, Alexander Whittam, Irvin Teh, Gustav Andersson, Fang-Cheng Yeh, Mikael Wiberg, Grainne Bourke

**Affiliations:** 1 Department of Plastic and Reconstructive Surgery, Leeds Teaching Hospitals Trust, Leeds, LS1 3EX UK; 2 Leeds Institute for Medical Research, University of Leeds, Leeds, UK; 3 Sheffield Teaching Hospitals NHS Foundation Trust, Sheffield, UK; 4 Leeds Institute for Cardiovascular and Metabolic Medicine, University of Leeds, Leeds, UK; 5 Department of Integrative Medical Biology, Faculty of Medicine, Umeå University, Umeå, Sweden; 6 Department of Surgical and Perioperative Science, Section for Hand and Plastic Surgery, Umeå University, Umeå, Sweden; 7 Department of Neurological Surgery, University of Pittsburgh, Pittsburgh, USA; 8 Wallenberg Centre for Molecular Medicine, Umeå University, Umeå, Sweden

**Keywords:** Diffusion tensor, Brachial plexus, Normal, Normative, Healthy, Peripheral nerve

## Abstract

**Purpose:**

Diffusion tensor magnetic resonance imaging (DTI) characterises tissue microstructure and provides proxy measures of myelination, axon diameter, fibre density and organisation. This may be valuable in the assessment of the roots of the brachial plexus in health and disease. Therefore, there is a need to define the normal DTI values.

**Methods:**

The literature was systematically searched for studies of asymptomatic adults who underwent DTI of the brachial plexus. Participant characteristics, scanning protocols, and measurements of the fractional anisotropy (FA) and mean diffusivity (MD) of each spinal root were extracted by two independent review authors. Generalised linear modelling was used to estimate the effect of experimental conditions on the FA and MD. Meta-analysis of root-level estimates was performed using Cohen’s method with random effects.

**Results:**

Nine articles, describing 316 adults (1:1 male:female) of mean age 35 years (SD 6) were included. Increments of ten diffusion sensitising gradient directions reduced the mean FA by 0.01 (95% CI 0.01, 0.03). Each year of life reduced the mean MD by 0.03 × 10^–3^ mm^2^/s (95% CI 0.01, 0.04). At 3-T, the pooled mean FA of the roots was 0.36 (95% CI 0.34, 0.38; *I*^2^ 98%). The pooled mean MD of the roots was 1.51 × 10^–3^ mm^2^/s (95% CI 1.45, 1.56; *I*^2^ 99%).

**Conclusions:**

The FA and MD of the roots of the brachial plexus vary according to experimental conditions and participant factors. We provide summary estimates of the normative values in different conditions which may be valuable to researchers and clinicians alike.

**Electronic supplementary material:**

The online version of this article (10.1007/s40336-020-00393-x) contains supplementary material, which is available to authorized users.

## Introduction

**Fig. 1:**
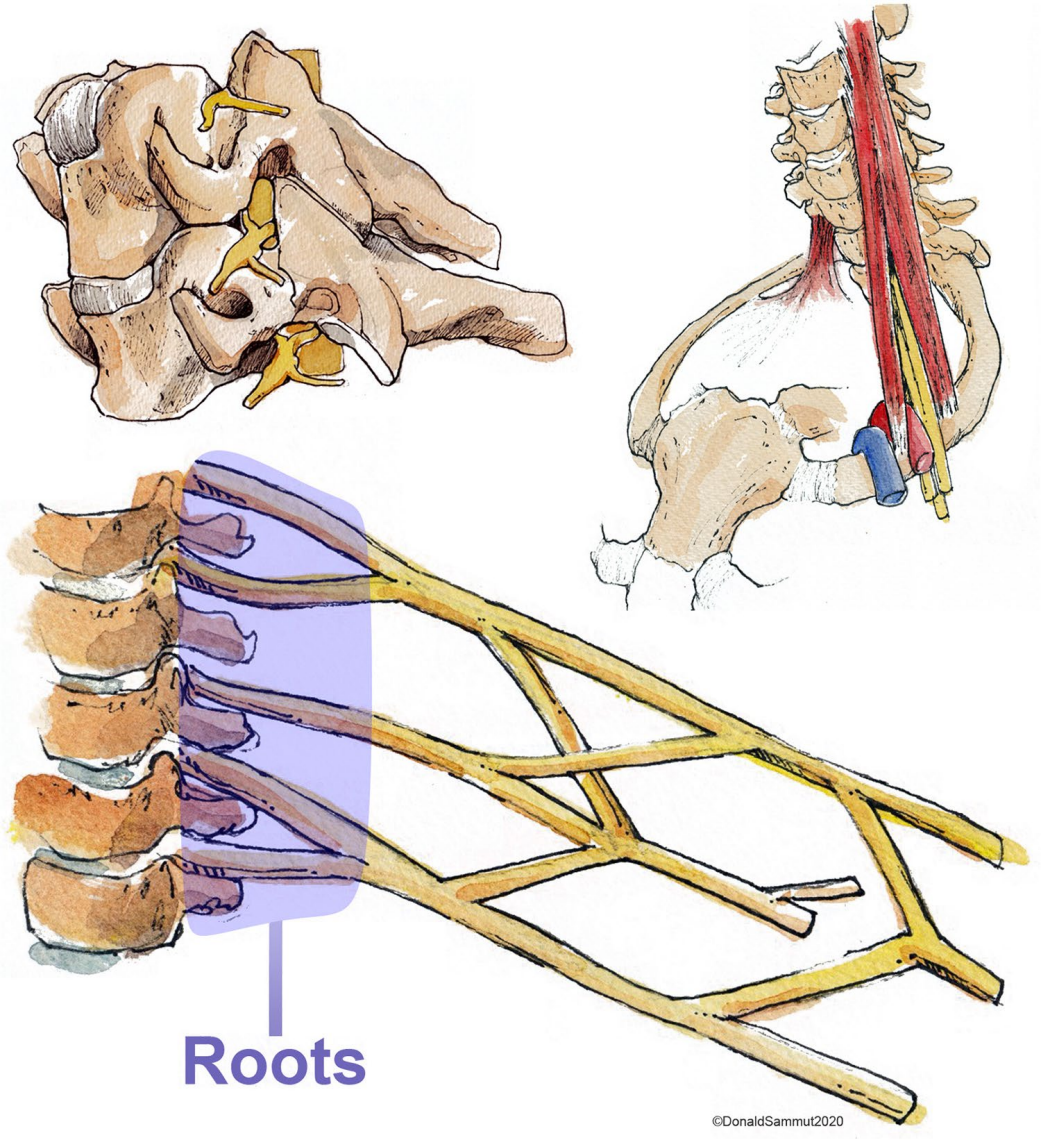
The roots of the brachial plexus emerging from the intervertebral foramina (upper left image) and their relationship to the scalene muscles and vasculature of the upper limb (upper right image). The lower image is a simplified schematic of the brachial plexus highlighting (in purple) the spinal roots. Reproduced with permission from Mr. Donald Sammut

The brachial plexus is a network of nerves which supply the upper limb with movement and feeling (Fig. 1 and Supplementary Fig. 1). Magnetic resonance imaging (MRI) is generally considered the best non-invasive imaging modality for diagnosing various pathologies affecting the brachial plexus [1–6]. The roots of the brachial plexus are the most common site of injury [7] and typically, the status of the root dictates the prognosis and surgical reconstruction. Consequently, defining the health status of the roots is of paramount importance. Whilst MRI is more accurate than electrophysiology [2, 6], ultrasonography [3–5, 8] and computed tomography myelography [9], the diagnostic performance of conventional cross-sectional MRI for assessing the spinal nerve roots remains suboptimal [7]. Consequently, there has been a surge of research into diffusion tensor imaging (DTI) which may provide additional valuable information. DTI characterises tissue microstructure and provides reproducible [10–13] proxy measures of nerve health which are sensitive to myelination, axon diameter, fibre density and organisation [14, 15]. The parameters typically derived from DTI include the fractional anisotropy (FA), mean diffusivity (MD), axial diffusivity (AD) and radial diffusivity (RD). FA is a scalar value between zero and one; an FA of zero implies isotropic diffusion within a voxel, whilst (in the context of peripheral nerve imaging) a FA nearing one implies diffusion along a single axis i.e., either anterograde of retrograde diffusion along the length of the nerve. MD describes the average molecular diffusion rate of the tensor; AD describes the diffusion rate in the long axis and RD represents diffusion perpendicular to the long axis.

Before researchers and clinicians can use DTI parameters to identify abnormal roots of the brachial plexus, there is a need to define the “normal” values. Numerous studies have examined aspects of DTI of the brachial plexus in healthy adults. This review aims to summarise the values observed in health and explore discrepancies in the reported measurements.

## Methods

This review is registered with PROPSERO (ID CRD42019155788), it was designed and conducted in accordance with the Cochrane Handbook of Systematic Reviews [16] and has been authored in accordance with the PRISMA checklist [17].

### Types of studies

We included all studies which reported the findings of diffusion tensor magnetic resonance imaging of the roots of the brachial plexus in healthy adults. Case reports were excluded.

### Participants

Asymptomatic adults (aged ≥ 16 years) with no known pathology (past or present) affecting the spinal cord or brachial plexus were the population of interest.

### Image acquisition

Studies must have reported diffusion tensor imaging parameters from the roots of the brachial plexus.

### Search strategy

The NICE Healthcare Databases (hdas.nice.org.uk) was searched using the terms “diffusion tensor” OR “DTI” AND “brachial plexus”. This yielded 67 hits in PubMed, 36 in Embase, 8 in CINAHL, 2 in CENTRAL and 2 in ClinicalTrials.gov the on 13th November 2019. After de-duplication, 78 unique citations were independently screened by two review authors (RGW and AW). The full texts of all potentially relevant articles were obtained. The reference lists for included articles were also scrutinised for potentially relevant papers. The final lists of included articles were compared and disagreements resolved by discussion.

### Study selection

Two review authors (RGW and AW) independently screened titles and abstracts for relevance, in accordance with the eligibility criteria. The full texts of potentially eligible articles were obtained and again independently assessed by the same two authors. Disagreements were resolved by discussion.

### Data extraction

Two review authors (RGW and AW) independently extracted data concerning the demographics, scanner, pulse sequence, pre-processing, tensorial reconstruction, measurement conditions and the outcomes of interest. The spinal nerve root was the unit of analysis [7] and root-level estimates of DTI parameters were extracted. Where data was missing or unclear, the corresponding author was contacted by email and/or phone and if no reply was received, 4 weeks later all authors were contacted in addition to re-contacting the corresponding author. The authors of one study [18] provided additional information (measurements form the extraforaminal roots using identical methods) for the purposes of this review.

### Outcomes

The primary outcome is to estimate the normal fractional anisotropy (FA) of the extraforaminal roots of the brachial plexus in healthy adults. The secondary outcomes include: (a) to estimate the normal mean diffusivity (MD) of the extraforaminal roots of the brachial plexus in healthy adults, and (b) to explore the associations between DTI parameters and participants age, the signal-to-noise (SNR) ratios and related factors such as the *b* value(s), echo time(s) (TE) and resolution (in cubic millimetres, mm^3^) and the number of diffusion sensitising gradient directions (N_D_) sampled per shell.

### Methodological quality assessment

The risk of bias was assessed by two review authors (RGW and AW) using the ROBINS-I tool [19] and displayed graphically using robvis [20]. Disagreements were resolved by discussion.

### Statistical analysis

Data were analysed in Stata/MP v15 (StataCop LLC, Texas). To estimate the effect of experimental/participant factors on the FA and MD, generalised linear modelling (GLM) was used with gaussian families. As the TE, *b* value and resolution are functions of SNR, the fixed effects were selected to be SNR, age in years and the *N*_D_ all of which were handled as continuous variables. The random effects in the GLM varied by the study. Estimates were bootstrapped using lossless non-parametric resampling with replacement, with 1000 iterations. There was insufficient data to meaningfully assess the effect of different tensor fitting methods or components of the *b* value (diffusion time, magnitude or interval) on DTI parameters. To visualise the association of FA with *N*_D_, and MD with age, scatterplots of the aggregate estimates were generated using the *metareg* package; the circles are root-level estimates and the sizes are dependent on the precision (inverse variance) of the estimate. To estimate the pooled normal FA and MD of the spinal roots, meta-analyses were performed using the *metan* package. Cohen’s method was used because both FA and MD were homoscedastic. Dersimonian and Laird random effects were used given the clinical heterogeneity. Analyses were subgrouped by both the N_D_ and spinal root (C5, C6, C7, C8, T1). Confidence intervals (CI) were generated to the 95% level. To assess the possibility of small-study effects we constructed a funnel plot using the *metafunnel* package, which is a scatterplot of the effect size against precision; symmetry implies the absence of small-study effects.

## Results

**Fig. 2:**
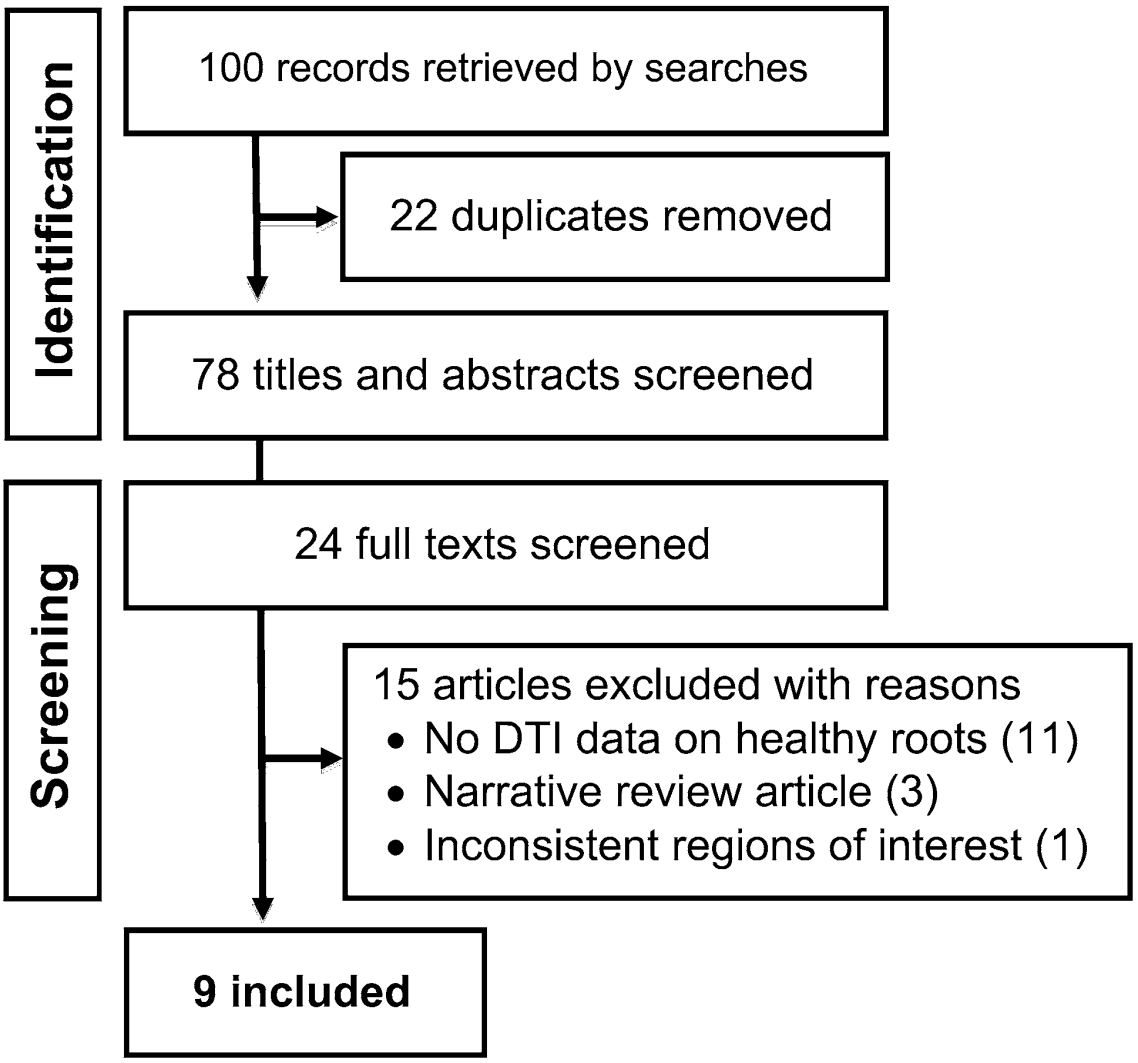
PRISMA flowchart

After reviewing 27 full texts, 15 were excluded (Supplementary materials) and 9 articles (of 9 unique studies) were included [18, 21–28] (Fig. 2).

### Study characteristics

Overall, 316 adults were included. The ratio of males:females was approximately 1:1 (156:154). The mean age of participants was 35 years (SD 6). The characteristics of included studies are shown in Table 1.

**Table 1:** Study characteristics

Population parameters				Scanner details		Data acquisition	DTI reconstruction		Measurement conditions			
Study	Location	N (M:F)	Age	Make and model	Field strength	Sequence	Pre-processing/corrections	Tensorial calculation	Mean SNR (SD)^a^	ROI position	ROI size	No. raters
Chen (2012) [23]	China	32	33.5	Philips Intera Master	1.5	ssEPI: b0/700/9001000 s/mm^2^, 32 directions, 2 averages, TE 77 ms, TR 9000 ms, 2 mm isotropic, axial slices	Not described	Not described	b700 = 18.3 (7.4) b900 = 17.0 (6.9) b1000 = 14.1 (5.1)	Not described	Not described	Not described
Ho (2017) [22]	Switzerland	10	30.6	Siemens Magnetom Skyra	3	ssEPI: b0/800 s/mm^2^, 30 directions, one acquisition (no averages), TE 67 ms, TR 5600 ms, 1.9 mm isotropic	﻿ Not described	Not described	Not described	Distal to the ganglia	11m^2^	2
Ho (2019) [21]	Switzerland	10	29.2	Siemens Magnetom Skyra	3	rsEPI: b0/900 s/mm^2^, 4 averages, TE 57 ms, TR 3030 ms, 2.4 mm isotropic, number of directions not described ssEPI: b0/900 s/mm^2^, 4 averages, TE 59 ms, TR 5800 ms, 2.4 mm isotropic, number of directions not described	Not described	Not described	rsEPI ≈ 6.5 (2) ssEPI ≈ 6.1 (2.4)	Distal to the ganglia	Bespoke	2
Oudeman (2018) [24]	Netherlands	30	44	Philips Ingenia	3	ssEPI: b0/800 s/mm^2^, 15 directions, TE 77 ms, TR 5969 ms, 6 averages, 3 mm isotropic	Correction for Rician noise, motion and eddy currents in DTItools	Weighted least linear squares	21 (8)	Close to the ganglia	Not described	2
Su (2019) [25]	China	163	38	Siemens Magnetom Aera	3	rsEPI: 0/900 s/mm^2^, 20 directions, 4 averages, TE 92 ms, TR 6000 ms, 2 × 2 × 3mm voxels	Not described	Not described	Not described	Postganglionic roots	Not described	2
Tagliafico (2011) [28]	Italy	40	44.5	GE, model not described	3	ssEPI: b0/1000 s/mm^2^, 32 directions, averaging not described, TE “minimum”, TR 16675 ms, 2 mm slice thickness	Correction for eddy currents using the General Electric Functool v6.3.1	Not described	Not described	Not described	2 mm^2^	4
Vargas (2010) [26]	Switzerland	12	41	Siemens Avanto	1.5	ssEPI: b0/900 s/mm^2^, 30 directions, TE 78 ms, TR 9000 ms, one acquisition (no averages), 2 mm isotropic, iPAT 2, axial slices	Not described	Not described	Not described	At the level of the proximal roots	2 mm^2^	3
Wade (2020) [27]	UK	10	28	Siemens Magnetom Prisma	3	ssEPI: b0/1000 s/mm^2^, 64 bipolar directions (twice refocused spin echo), TE 66 ms, TR 4300 ms, 8 averages (4 per phase encoding direction), 2.5 mm isotropic, 2nd order in-line motion correction, axial slices	Correction for susceptibility artefacts in DSI Studio	Linear least squares	67.6 (45)	Extraforaminal roots, 3 cm lateral to the midline of the spinal cord	5 mm^3^ (8 voxels)	2
Wade (2020) [18]	UK	7	28	Siemens Magnetom Prisma	3	ssEPI: b0/1000 s/mm^2^, 20 monopolar directions, 4 averages, TE 66 ms, TR 4300 ms, 2.5 mm isotropic, second order in-line motion correction, axial slices	None	Linear least squares	36.0 (16)	Extraforaminal roots, 3 cm lateral to the midline of the spinal cord	5mm^3^ (8 voxels)	2

*ssEPI* single-shot echo-planar imaging, *rsEPI* readout segment echo-planar imaging

^a^Study-level signal-to-noise ratio (SNR) derived from non-diffusion-weighted (b0) images

### Risk of bias within studies

**Fig. 3:**
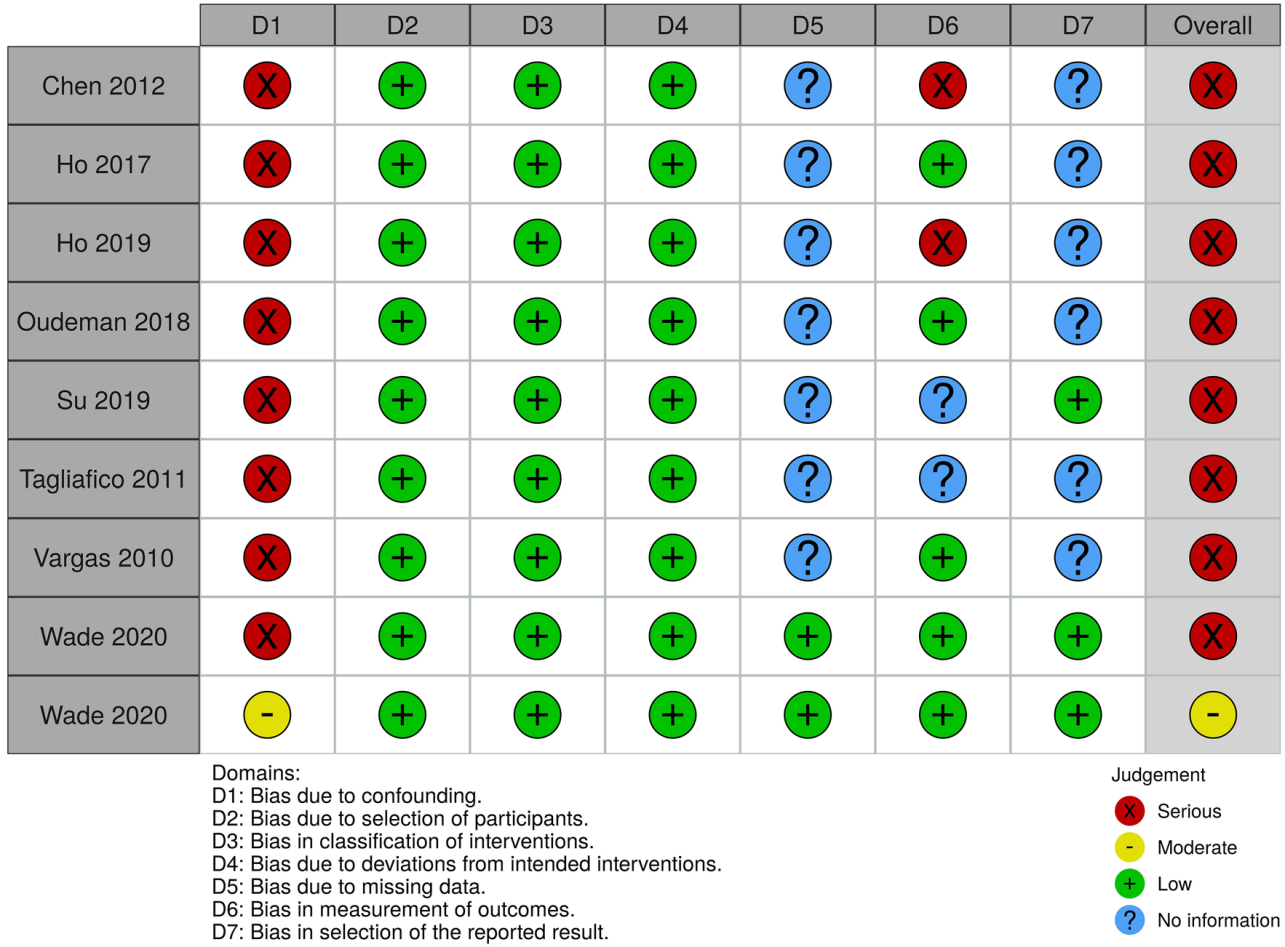
The summary risk of bias plot for included studies. Red = high risk, yellow = unclear risk, green = low risk

The risk of bias for the included studies are summarised in Fig. 3. Eight of the included studies [18, 21–24, 26, 28, 29] were at risk of bias due to confounding because there was no adjustment for a) effect modifiers such as age, weight and the experimental factors described above, and b) repeated measures, i.e. ten estimates of the FA (one from each spinal root, bilaterally) taken from the same individual will be highly correlated and without adjustment, the sample-level estimates will have falsely small variances. Seven studies [21–25, 28] provided no information about missing data and so the risk of bias is unclear. Two studies [21, 23] were judged to be at high risk of bias in the measurement of FA and MD because a bespoke region of interest was used to calculate the FA and the number of diffusion sensitising gradients used was not described [21]. Three studies [24, 25, 28] were at unclear risk of bias in the measurement of FA and MD because information was lacking about pre-processing, tensorial reconstruction or how the estimates of the FA and MD were derived from images (e.g. region of interest size and position). One study [23] was at high risk of reporting bias because three *b* values were tested (700, 900, 1100 mm/s) but it is unclear which yielded the estimates of FA and MD reported in the manuscript or whether they are an average of the three. The risk of bias due to selective outcome reporting was unclear in six studies [21–24, 26, 28], because no information was provided regarding the exclusion criteria, attrition due to scan intolerance, dataset exclusion (e.g. for uncorrectable motion artefact) or otherwise and there were no published protocols to consult.

### Evidence synthesis

**Fig. 4:**
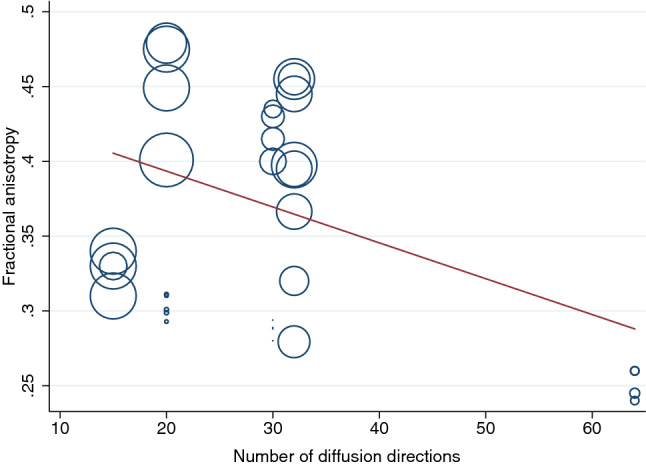
A scatterplot showing the negative association between the mean fractional anisotropy of the roots of the brachial plexus and the number of diffusion sensitising gradient directions

**Fig. 5:**
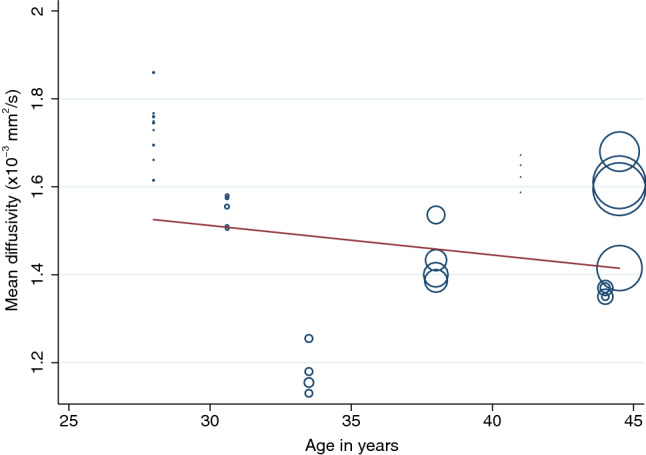
A scatterplot showing the negative association between the mean diffusivity of the roots of the brachial plexus and the mean age of adults in the included studies

There were no clear associations between experimental factors and the FA or MD on univariable modelling (Table 2). Multivariable modelling showed that the angular resolution was strongly associated with FA, whereby every additional ten diffusion sensitising gradient directions sampled reduced the FA by 0.01 (95% CI 0.01, 0.03; Fig. 4). Furthermore, multivariable modelling showed that each year of life reduced the MD by 0.03 × 10^–3^ mm^2^/s (95% CI 0.01, 0.04; Fig. 5). Bootstrapping did not change these estimates.

**Table 2:** Mixed-effects generalised linear modeling showing the unadjusted, multivariable and bootstrapped multivariable effect estimates of co-variables on fractional anisotropy and mean diffusivity of the roots of the brachial plexus

DTI parameter	Experimental factors	Unadjusted coefficients (95% CI)	*p* value	Adjusted coefficients (95% CI)	*p* value	Resampled adjusted coefficients (95% CI)	Resampled *p* value
Fractional anisotropy	SNR	0.0001 (− 0.0003, 0.0004)	0.662	0.0001 (− 0.0001, 0.0004)	0.257	0.0001 (− 0.001, 0.002)	0.840
	Age in years	0.0003 (− 0.006, 0007)	0.926	0.001 (0.001, 0.002)	0.001	0.001 (− 0001, 0.003)	0.236
	Number of diffusion directions	− 0.002 (− 0.005, 0001)	0.183	− 0.001 (− 0.002, − 0.001)	< 0.001	− 0.001 (− 0.002, − 0.0004)	0.002
	Echo time (ms)	0.002 [0.00001, 0.004])	0.174	^a^	^a^	^a^	^a^
	*b* value (mm/s)	− 0.0004 (− 0.001, 0.0001)	0.098	^a^	^a^	^a^	^a^
	Resolution (mm^3^)	0.008 (0.003, 0.013)	0.126	^a^	^a^	^a^	^a^
Mean diffusivity (mm^2^/s × 10^–3^)	SNR	− 0.001 (− 0.003, 0.0005)	0.154	− 0.001 (− 0.003, 0)	0.055	− 0.001 (− 0.012, 0.01)	0.799
	Age in years	− 0.01 (− 0.03, 0.01)	0.303	− 0.025 (− 0.030, − 0.021)	< 0.001	− 0.03 (− 0.042, − 0.01)	0.003
	Number of diffusion directions	0.005 (− 0003, 0013)	0.234	0.001 (− 0.001, 0.002)	0.238	0.001 (− 0.005, 0.006)	0.723
	Echo time (ms)	− 0.012 (− 0.019, − 0.005)	0.174	^a^	^a^	^a^	^a^
	*b* value (mm/s)	0.001 (0.0004, 0.002)	0.003	^a^	^a^	^a^	^a^
	Resolution (mm^3^)	0.004 (0.002, 0.007)	0.562	^a^	^a^	^a^	^a^

^a^Excluded due to multicollinearity

**Fig. 6:**
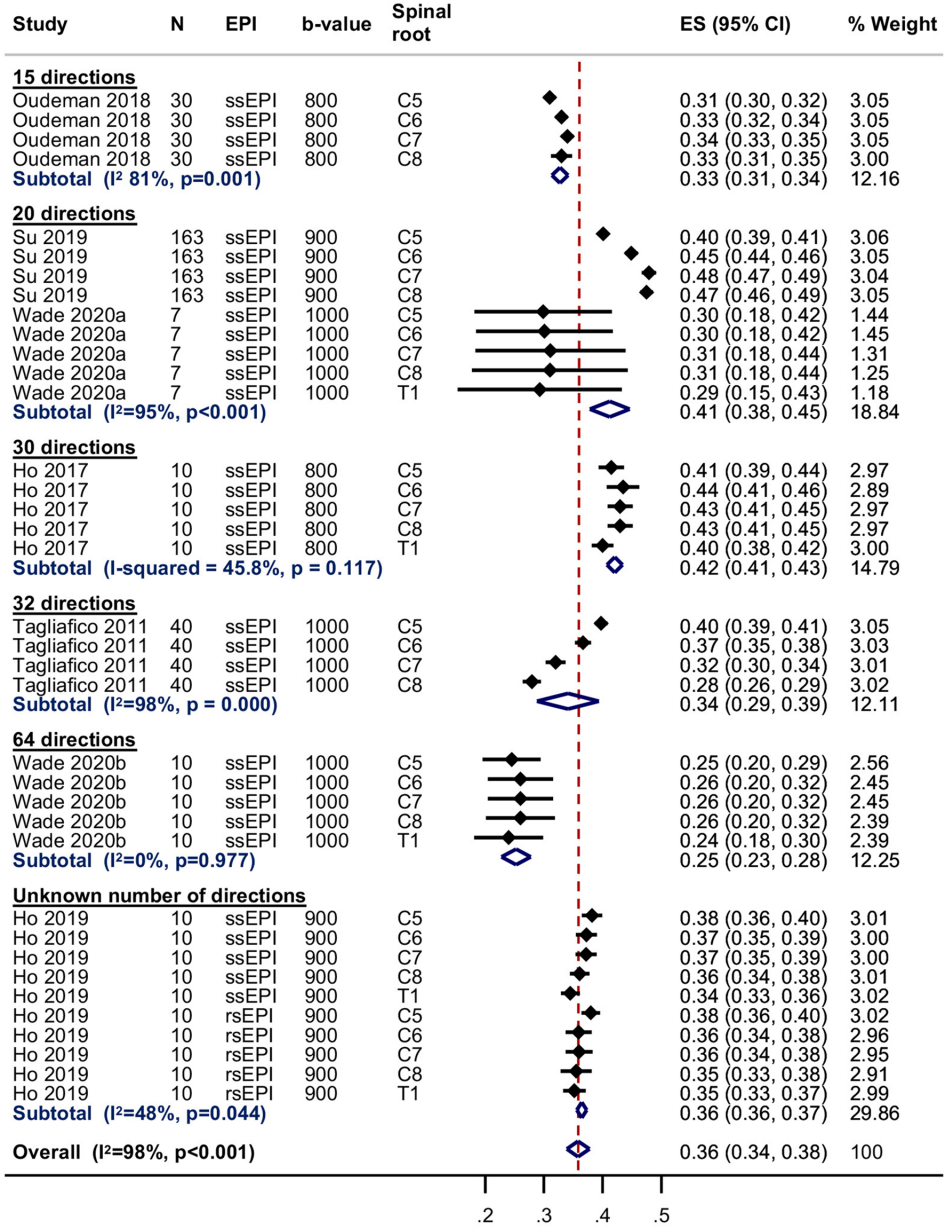
Summary estimates of the normal fractional anisotropy of the roots of the brachial plexus at 3-T

Seven studies [18, 21, 22, 24, 27–29] were included in the meta-analysis of the normal FA of the roots of the brachial plexus at 3 T; one study [26] did not provide estimates of the variance so could not be included. The pooled estimate of the normal FA of the root was 0.36 (95% CI 0.34, 0.38; Fig. 6). There were no statistically significant differences between the five roots. However, there was significant statistical heterogeneity between studies (*I*^2^ 98%) which may be related to the experimental conditions described above. The pooled estimates of the normal FA subgrouped by spinal root (including the study performed at 1.5 T which had usable data [23]) are shown in Supplementary Fig. 2.

**Fig. 7:**
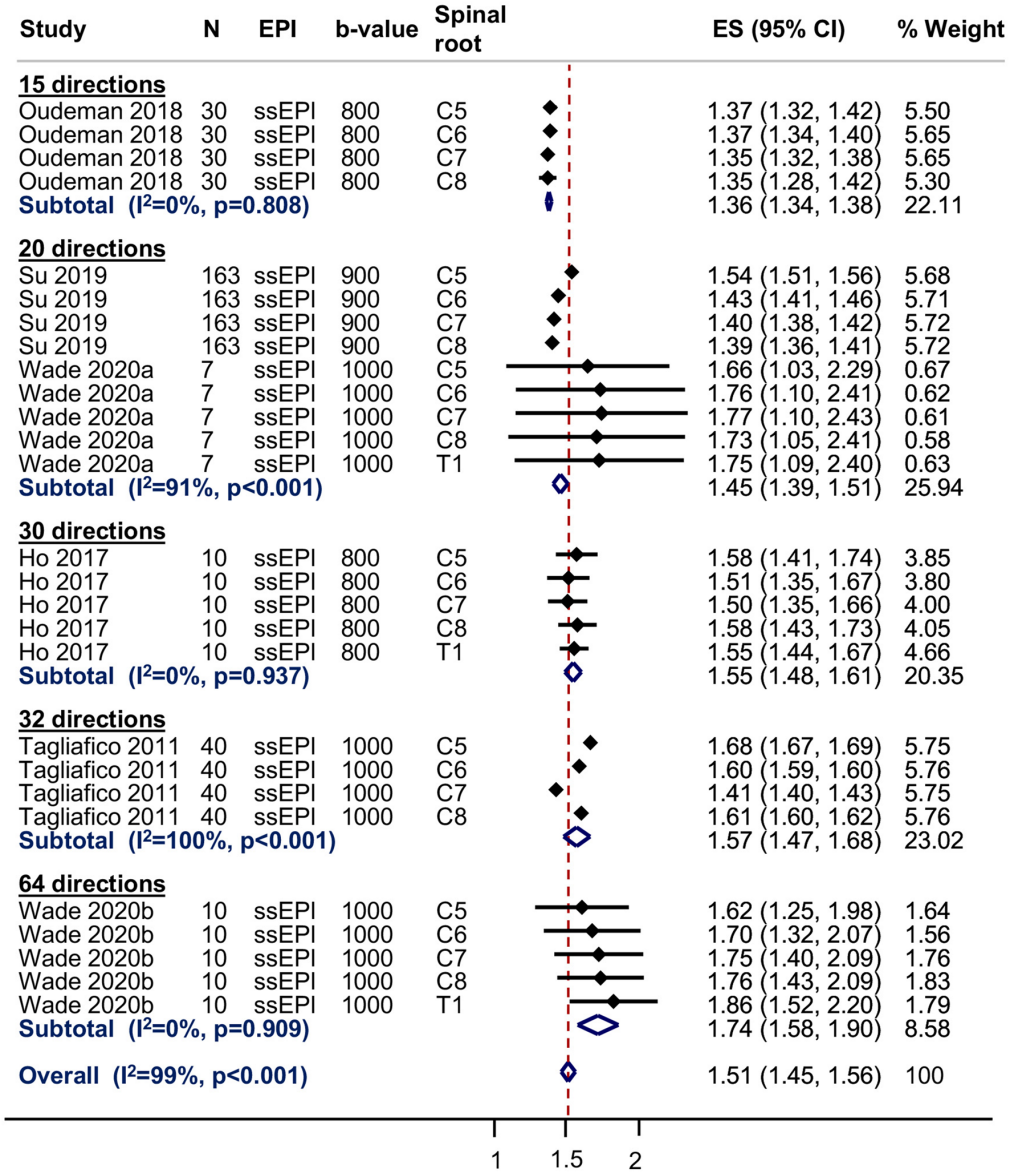
Summary estimates of the normal mean diffusivity of the roots of the brachial plexus at 3-T

Six studies [18, 22, 24, 27–29] contributed to the meta-analysis of the normal MD of the roots of the brachial plexus at 3 T; one study [26] did not provide estimates of the variance and one study [21] did not report the MD of any roots. The pooled estimate of the normal MD of the roots of the brachial plexus was 1.51 × 10^–3^ mm^2^/s (95% CI 1.45, 1.56; Fig. 7 and Supplementary Fig. 3); however, there was high statistical heterogeneity between studies (*I*^2^ 99%) which may be related to the experimental conditions described above. The pooled estimates of the normal MD subgrouped by spinal root (including the study performed at 1.5 T which had usable data [23]) is shown in Supplementary Fig. 3.

**Fig. 8:**
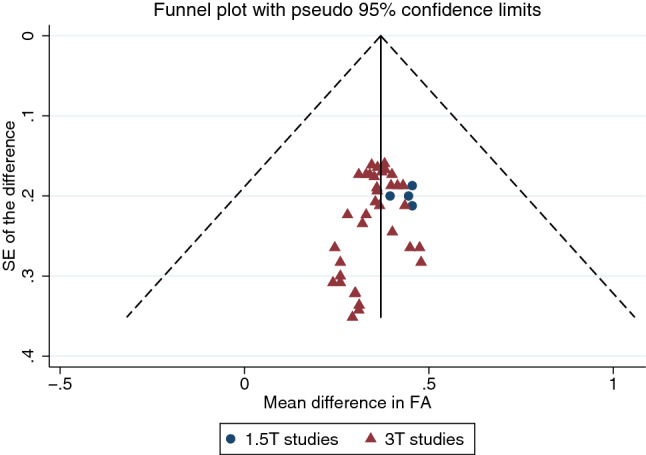
A funnel plot showing no evidence of small-study effects

There was no evidence of small-study effects (Fig. 8).

## Discussion

We have shown that the roots of the brachial plexus in adults have a mean fractional anisotropy of 0.36 (95% CI 0.34, 0.38) and mean diffusivity of 1.51 × 10^–3^ mm^2^/s (95% CI 1.45, 1.56). However, there is substantial variation and estimates are affected by experimental conditions such as the field strength, b value and N_D_ as well as patient factors, such as age.

Although we provide no direct comparisons, this work reinforces the generally accepted concept that the DTI parameters of the peripheral nervous system are different to those of the central white matter tracts of the brain [27]. The systematic reviews by Label [30] and Yap [31] showed that in health, the white matter tracts of the brain have a mean FA of approximately 0.36–0.54 and mean MD of 0.84–1.43 × 10^–3^ mm^2^/s, depending on the fibre bundle measured and the age of the individual. We find that the roots of the brachial plexus have a substantially lower average FA (between 0.34 and 0.38 with 95% confidence) and higher MD (95% CI 1.45–1.56 mm^2^/s × 10^–3^), which are comparable to the lower bounds of the central white matter values. We believe that these discrepancies are likely to be related to differences in the axon density (the corpus callosum has 38,000 myelinated fibres per mm^2^, whereas the brachial plexus has approximately 8348 fibres per mm^2^) [32, 33] and extensive intraneural fascicular sharing/crossing [34, 35].

Several experimental factors [36] are known to affect DTI parameter estimates, including: (a) scanning conditions such as the SNR [37], components of the *b* value [38, 39], *N*_D_ [40, 41]; (b) software pipelines for denoising, correcting artefacts arising from susceptibility, motion and eddy currents [42, 43] and tensor fitting methods [37], as well as (c) the size and position of ROIs which may contribute to partial volume effects [44]. Despite these limitations, the TraCED challenge [10] and several phantom studies [11–13] have demonstrated very high reproducibility across scanners, sequences and sessions for tractography from DTI. With more sophisticated diffusion sequences, the reasons for the disparities between central and peripheral fibre diffusion parameters may become apparent. Nonetheless, we have shown DTI parameters (and thus, probably, tractograms derived from these datasets) are related to numerous experimental conditions and, therefore, we suggest that researchers and clinicians interpret our summary values with both caution and respect to their particular circumstances.

In this study, the *N*_D_ was strongly related to the FA whereby fewer directions were associated with higher estimates of the FA. This is a well-known phenomenon [41] and likely to be explained by the association between noise (which can couple to give rise to anisotropy) and artefactually high estimates of the FA [37]. Whilst the observed FA values in the studies with relatively fewer directions and lower SNR appear plausible (rather than e.g. 0.9 which would clearly be artefactual), it is possible that such studies are more susceptible to bias and their estimates of FA are falsely high. Equally, our model may still be subject to collinearity because studies with higher *N*_D_ tended to also have a higher SNR. Conversely, as MD measures the size of the diffusion ellipsoid [45], we expected [41] MD to be independent of the N_D_ and this is what we observed.

As humans age, axons lose their integrity, peripheral nerves demyelinate and there is a corresponding increase in extra-cellular fluid. Consequently, advancing aging is typically associated with reduced FA and increased diffusivity in white matter structures [31, 46]. Prior work by Kronlage et al. [47] on the peripheral nerves in the forearm showed that the FA reduced with age (as in the brain [31]). We observed no statistically significant association between age and the FA of the roots of the brachial plexus but this may be due to the narrow age range of participants in our study. Whilst Kronlage [47] found that MD increased with age, we found that MD was slowed by 0.03 × 10^–3^ mm^2^/s with each year of life (Fig. 5). Our findings are in agreement with the wider literature on age-related diffusivity changes in the brain [46] and compatible with the biological mechanisms of aging. Specifically, we observed that the MD of the roots of the brachial plexus slowed in the 3rd and 4th decade of life, which has also been observed in the healthy senescent adult brain [46]. It is unclear why Kronlage’s work differs but this might be due to discrepancies in the age range of the sample (adults in this review were aged 28–45 years versus 20–80 years in Kronlage’s [47]) and aspects of the scanning because Kronlage [47] used non-isotropic voxels (4.0 mm through plane, 1.5 mm^2^ in plane) which might underestimate the FA and overestimate the MD [43].

### Limitations

The main limitation of this review is the pooled estimation of FA and MD. We decided to perform meta-analyses in the presence of high statistical heterogeneity because (a) the generated outputs provide an important graphical representation of the variability of measurements in relation to experimental conditions which are easy to interpret, and (b) the forest plots provide a pictorial representation of the deleterious effects of failing to adjust study-level estimates for repeated measures. Figures 6 and 7 and Supplementary Figs. 2 and 3 show that in the eight studies [18, 21–24, 26, 28, 29] which did not use multilevel models, the standard errors of the mean (and thus, their CIs) are falsely small. We believe that if studies had appropriately adjusted for clustering/repeated measures, the CIs would be wider, so overlaping the aggregate means from other studies and the measures of statistical heterogeneity (e.g. *I*^2^) would fall.

We show a negative association between MD and age; however, readers should note that the range of aggregate ages in the included studies is narrow (28–45 years) and so the estimates may not be generalisable to the population.

## Conclusions

The roots of the brachial plexus in adults appear to have a pooled mean fractional anisotropy of 0.36 (95% CI 0.34, 0.38) and pooled mean diffusivity of 1.51 × 10^–3^ mm^2^/s (95% CI 1.45, 1.56), although these parameters are dependent on experimental conditions and vary slightly from C5 to T1.

## Electronic supplementary material

Below is the link to the electronic supplementary material.

Supplementary file1 (DOCX 650 kb)

## Data Availability

The extracted data and statistical syntax are available from the first author upon request.
