# Supplementary material for: Diffusion tensor imaging of the roots of the brachial plexus: a systematic review and meta-analysis of normative values
Source: Clin Transl Imaging. 2020 Oct 9;8(6):419–31. doi: 10.1007/s40336-020-00393-x (PMC7708343; doi:10.1007/s40336-020-00393-x)
Supplement: Supplementary file 1 — Supplementary file1 (DOCX 650 kb) [file 40336_2020_393_MOESM1_ESM.docx]

**Supplementary Figure 1.** A schematic of the of the anatomy of the brachial plexus courtesy of Donald Sammut (<https://www.donaldsammut.com/>)

**
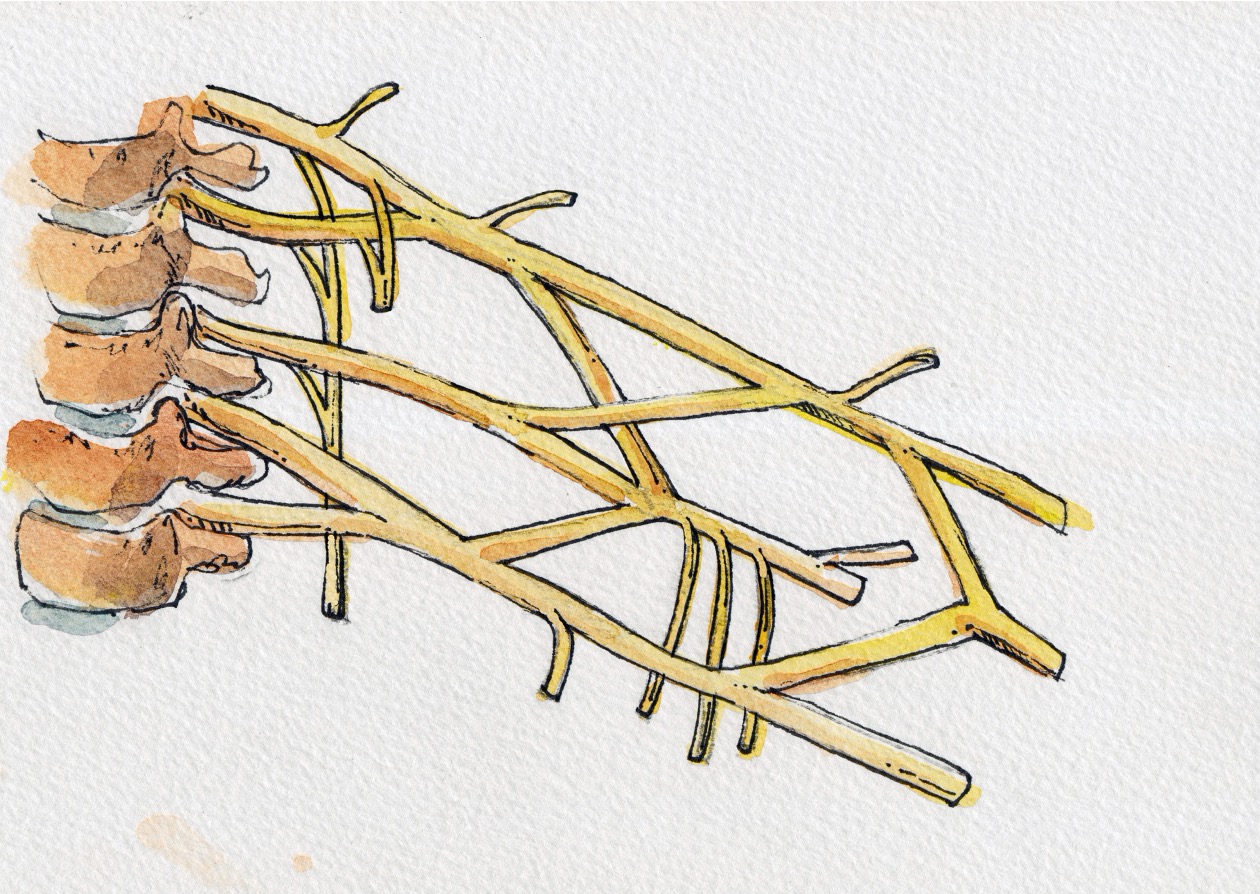
**

**Supplementary Figure 2.** Summary estimates of the normal fractional anisotropy of the roots of the brachial plexus, subgrouped by cervical root.

**
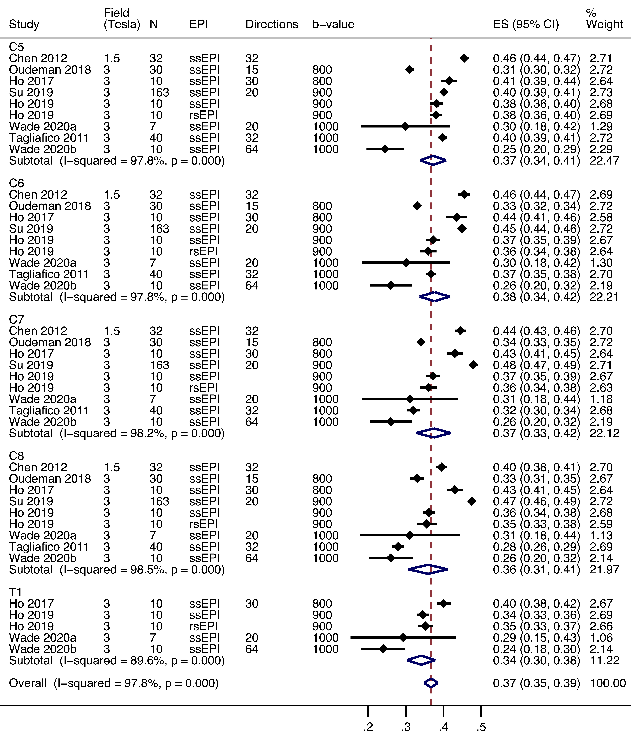
**

**Supplementary Figure 3.** Summary estimates of the normal mean diffusivity of the roots of the brachial plexus, subgrouped by cervical root.

**
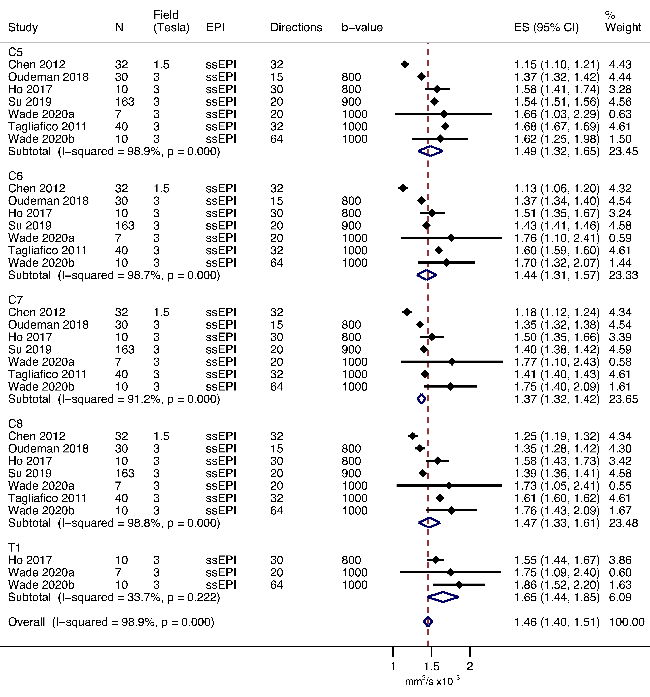
**

**Excluded studies**

Evaluation of Brachial Plexus Using Combined Stereological Techniques of Diffusion Tensor Imaging and Fiber Tracking. 2019. Acer N and Turgut M. Journal of brachial plexus and peripheral nerve injury. e16-e23. 14. 10.1055/s-0039-1687913

Zobrazeni perifernich nervu pomoci difuzniho tenzoru a mr traktografieImaging of peripheral nerves using diffusion tensor imaging and MR tractography. 2018. Humhej I. et al. Ceska a Slovenska Neurologie a Neurochirurgie. 420-426. 81. 4. 10.14735/amcsnn2018csnn.eu3

Diffusion tensor imaging of the brachial plexus as an aid to the diagnosis of inflammatory neuropathies: Preliminary results. 2016. Oudeman J. et al. Journal of the Peripheral Nervous System. 198. 21. 3. 1529-8027. Blackwell Publishing Inc.

New technologies for the assessment of neuropathies.2017. Gasparotti R, et al. Nature Reviews Neurology. 203-216. 13. 4. 10.1038/nrneurol.2017.31

Diffusion tensor imaging of the brachial plexus and cervical spinal nerve roots: Case control study in CIDP patients and controls.2017. Chanson E, et al. Neurology. 88. 16. 1526-632X

Clinical application of diffusion tensor tractography (DTT) of traumatic brachial plexus injuries. 2011. Schmidt T, et al. Journal of Neurosurgery. 115. 2. 0022-3085. American Association of Neurological Surgeons

Mri of the brachial plexus and chronic inflammatory demyelinating polyradiculoneuropathy: assessment of dti-derived measurements at 3.0-t. 2017. Chanson E, et al. Journal of the Peripheral Nervous System. 226-414. 22. 3. 10.1111/jns.12225

Feasibility of diffusion tensor tractography of brachial plexus injuries at 1.5 T. 2013. Gasparotti R, et al. Investigative Radiology. 104-112. 48. 2. 10.1097/RLI.0b013e3182775267

Quantitative MR neurography of brachial plexus lesions based on diffusivity measurements

2018. Hend Galal Eldeen et al. The Egyptian Journal of Radiology and Nuclear Medicine. 1093-1102

49. 4. 10.1016/j.ejrnm.2018.05.005

Quantitative magnetic resonance (MR) neurography for evaluation of peripheral nerves and plexus injuries. 2017. Noguerol M, et al. Quantitative imaging in medicine and surgery. 398-421. 7. 4

3T MR tomography of the brachial plexus: Structural and microstructural evaluation. 2012. Mallouhi A, et al. European Journal of Radiology. 2231-2245. 81. 9. 10.1016/j.ejrad.2011.05.021

High-resolution and functional magnetic resonance imaging of the brachial plexus using an isotropic 3D T2 STIR (Short Term Inversion Recovery) SPACE sequence and diffusion tensor imaging

2008. Viallon M, et al. European Radiology. 1018-1023. 18. 5. 10.1007/s00330-007-0834-4

Nervenverletzungen und traumatische Läsionen des Plexus brachialis. 2017. 1. Schwarz D, et al. Der Radiologe. 184-194. 57. 3. 10.1007/s00117-017-0207-1

“Diffusion tensor imaging and tractography of traumatic brachial plexus palsies” presented at the 38th european society of neuroradiology Diagnostic and Interventional annual meeting. 2015. Gasparotti R, et al. Neuroradiology. 1-169. 57. S1. 10.1007/s00234-015-1557-x

“Diffusion tensor imaging and tractography of traumatic brachial plexus palsies. Preliminary experience”. 2010. Gasparotti R, et al. Neuroradiology Journal. 361. 23. 1971-4009

DTI of brachial plexus. 2010. Pellicano G, et al.Neuroradiology Journal. 80. 23. 1971-4009

Cerebral plasticity in painful phantom limb: a study with transcranial magnetic stimulation and functional magnetic resonance imaging. 2009. de Oliveira, et al. European Journal of Pain. S70. 13. 10.1016/S1090-3801(09)60218-4

Visualizing nerve fibers surrounding a brachial plexus tumor using MR diffusion tensor imaging

no normal DTI data. Gallagher TA, et al. Neurology. 2016. 65. 582-583. 10.1212/WNL.00000000000023602016
